# Voxelated opto-physically unclonable functions via irreplicable wrinkles

**DOI:** 10.1038/s41377-023-01285-1

**Published:** 2023-10-03

**Authors:** Kitae Kim, Se-Um Kim, Moon-Young Choi, Mohsin Hassan Saeed, Youngmin Kim, Jun-Hee Na

**Affiliations:** 1 Department of Convergence System Engineering, Chungnam National University, 99 Daehak-ro, Yuseong-gu, Daejeon 34134 Republic of Korea; 2 Department of Electrical and Information Engineering, Seoul National University of Science & Technology, 232 Gongneung-ro, Nowon-gu, Seoul 01811 Republic of Korea; 3 Department of Electrical, Electronics, and Communication Engineering Education, Chungnam National University, 99 Daehak-ro, Yuseong-gu, Daejeon 34134 Republic of Korea; 4 Hologram Research Center, Korea Electronics Technology Institute, World Cup buk-ro 54-gil, Mapo-gu, Seoul 03924 Republic of Korea

**Keywords:** Optical data storage, Photonic devices

## Abstract

The increased prevalence of the Internet of Things (IoT) and the integration of digital technology into our daily lives have given rise to heightened security risks and the need for more robust security measures. In response to these challenges, physical unclonable functions (PUFs) have emerged as promising solution, offering a highly secure method to generate unpredictable and unique random digital values by leveraging inherent physical characteristics. However, traditional PUFs implementations often require complex hardware and circuitry, which can add to the cost and complexity of the system. We present a novel approach using a random wrinkles PUF (rw-PUF) based on an optically anisotropic, facile, simple, and cost-effective material. These wrinkles contain randomly oriented liquid crystal molecules, resulting in a two-dimensional retardation map corresponding to a complex birefringence pattern. Additionally, our proposed technique allows for customization based on specific requirements using a spatial light modulator, enabling fast fabrication. The random wrinkles PUF has the capability to store multiple data sets within a single PUF without the need for physical alterations. Furthermore, we introduce a concept called ‘polyhedron authentication,’ which utilizes three-dimensional information storage in a voxelated random wrinkles PUF. This approach demonstrates the feasibility of implementing high-level security technology by leveraging the unique properties of the rw-PUF.

## Abstract (web summary)

The study introduces a novel approach to information encoding using 2D random wrinkles PUFs in a 3D framework, utilizing polarization manipulation and data extracting functions to enhance security in authentication systems.

## Graphical abstract

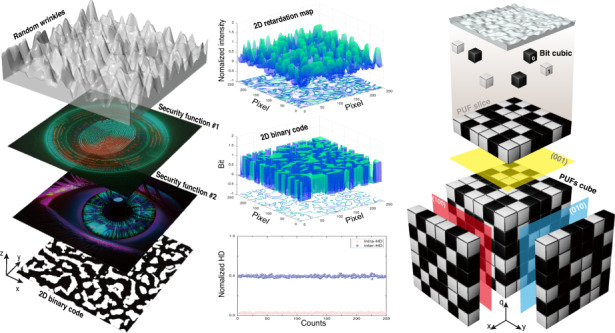

## Introduction

With the integration of digital technology and the internet, complex digital networks have emerged, utilizing ubiquitous electronic devices such as mobile phones and smart appliances. This has enabled the creation of various services and usage environments, including financial transactions. As a result, our information can be accessed and exchanged anywhere, using technologies such as the Internet of Things (IoT), big data, and cloud computing^1–7^. However, this information technology environment is also prone to security breaches such as personal information leakage and cyber-attacks, which require the implementation of a robust and secure security system. In addition, security concerns related to existing software-based digital encryption necessitate more secure and non-replicable hardware-based security^8–13^. Although several security systems, such as firewalls and biometric authentication, have been developed, they have not kept up with increasingly sophisticated hacking methods. Therefore, the development of a highly robust security system is essential to address these fundamental challenges.

Physical unclonable functions (PUFs) are a technology that has unique values, such as human fingerprints and iris^13^. It is a system that generates random digital values that are difficult to predict by utilizing characteristics formed by process deviations that occur in semiconductor process characteristics. A general software-based security technique generates random numbers in an external computer program and stores them in a non-volatile memory (NVM) inside a device^14,15^. The recorded keys are always at risk of leakage and vulnerable security. However, the PUF has a random number on its own, and the critical value is self-generated without having to inject the random number value separately from the outside, which significantly reduces the leakage risk. PUF is a random value in itself^16–18^. The PUF is divided into mismatch-based PUF and physical-based PUF, depending on the implementation method. Mismatch-based PUF uses the fact that the characteristics differ depending on the variation of the device manufacturing process. Typically, an Arbiter PUF uses different signal delay times caused by process deviation. A physical-based PUF is a method of determining a PUF value using the physical characteristics of the device. In addition, a physical-based PUF is a method of determining the PUF value using the physical characteristics of the device, and there is a via-PUF that generates a random value based on the probability of forming through silicon via (FSV) in the semiconductor.

Furthermore, there are circuit-based XOR PUFs^19^, Feed-Forward PUFs^20–22^, Ring oscillator PUFs^17,23,24^, memory-based SRAM PUFs^25^, Latch PUFs^13^, and optical PUFs^8,26–31^ that detect and utilize optical signals. Recently, research has been reported on the development of optical PUFs that use chemical and material technologies such as luminescent nano/microparticles to generate optical signals^32,33^ and utilize transmission, reflection, diffraction light by surface microstructure^34–36^, and optical anisotropic materials^37,38^. However, the conventional optical PUF is challenging to apply in practical use due to a complex organic molecule^39^, the short life of fluorescent molecules^34^, and a high sensitivity to the surrounding environment, such as ambient light intensity and temperature.

Here, we present a stereoscopic opto-physical unclonable function (PUF) based on a unique birefringent wrinkled structure. In our previous research, we demonstrated that surface wrinkles could be produced in desired shapes using reactive mesogens (RMs) and a spatial light modulator (SLM). These wrinkled structures have potential applications in anti-counterfeiting technology, information storage, and image labeling^40–42^. RMs are an optically anisotropic medium that can produce a polymer network with uniform orientation via photopolymerization through a reactor, making them useful in various optical applications^43^. We achieve a highly random nature of non-aligned wrinkles for optical PUF applications, which can produce an inimitable pattern through diffraction by the birefringence characteristics of RM. Unlike conventional laser-based methods, diffraction-based pattern images can be easily and quickly obtained using visible light. Furthermore, we demonstrate using the birefringence pattern generated by the random wrinkle physical unclonable function (rw-PUF) as a highly secure authentication key. The random wrinkles PUF holds potential for applications beyond authentication systems, including anti-counterfeiting and data storage technology.

## Results

### Opto-physical PUF using random wrinkles of anisotropic medium

**Fig. 1:**
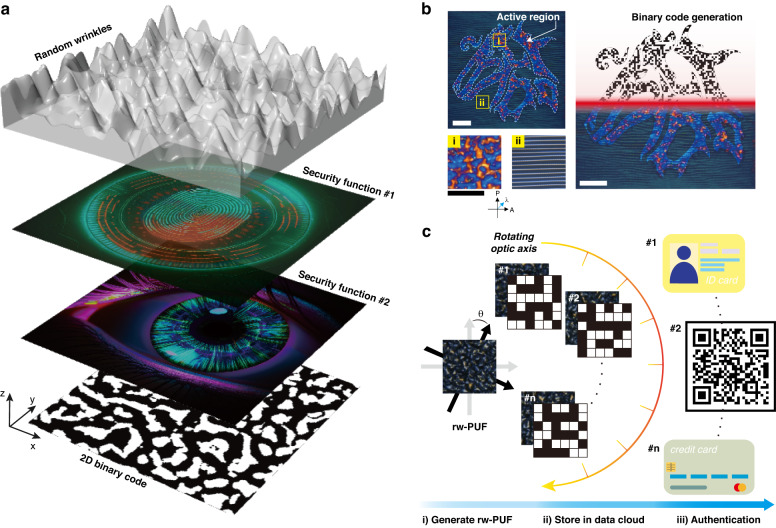
Voxelated random wrinkles of physical unclonable function (PUF). **a** Schematic illustration of encoded information stored in random wrinkles with spatial optical retardation varying caused by birefringence of the anisotropic medium. Random anisotropic wrinkles can generate an infinite number of security codes which are 2D binary codes. **b** Unstandardized security patterns can be implemented, and grayscale can be adjusted based on the retardation of each individual pixel that comprises the pattern. When passing through a wave plate (*λ* = 530 nm), the retardation can be observed as blue- and red-shifted pixels. The resulting wrinkles can then be transformed into a distinctive binary code through image processing. All scale bar represents 50 μm. **c** Overall rw-PUF data encryption and application through the following process: (i) The rw-PUF is generated through a random wrinkling process. (ii) The pixelated retardation from a single rw-PUF, formed by the angle to the crossed polarizer, is encrypted and stored in the data cloud. (iii) The stored data is decrypted and authenticated as required for the application

RMs comprise a mesogen core and one or two acrylates that respond to ultraviolet (UV) light. The acrylates act as a reactor upon appropriate UV irradiation to facilitate photopolymerization and form an optically anisotropic stationary phase. The uniformity of the optically anisotropic wrinkles formed by these reactive mesogens depends on the accuracy of their orientation. When the orientation force is vital in the same direction, the wrinkles are uniformly formed in a single direction, leading to uniform optical diffraction patterns such as grating. In contrast, insufficient orientation force results in a non-uniform wrinkle direction, leading to random absorption, reflection, and diffraction of light passing through the surface. The optical characteristics of these wrinkles can be utilized to create a unique security pattern. Our previous research demonstrated that the direction of light transmitted through these wrinkles between crossed polarizers varies depending on their direction^40^. Based on this notion, by adjusting the orientation of the crossed polarizers, the intensity of the light passing through the disordered wrinkles can also be changed. Figure 1a depicts the conceptual schematic of encoding information in a single PUF. The orientation of molecules within a material can impact light transmission through it. Wrinkles formed between crossed polarizers can influence the direction-dependent transmittance of light, where the angle of the cross-polarizer relative to the wrinkles is referred to as the ‘optic axis’. When this optic axis changes, the transmission of light through the random wrinkles becomes unpredictable, which can be utilized to create physical unclonable codes. These codes are valuable for data storage and identification. A single optical PUF can produce multiple result values by manipulating the optic axis, providing an alternative to diverse data such as fingerprints and iris recognition. The optical setup used in this study employs a SLM to direct polarized UV light to a specific region of the photoalignment layer, allowing for the alteration of the direction of reactive mesogens upon contact with the layer. By creating a region without contact with polarized UV, wrinkles with random directions can be formed in desired patterns. The alignment of liquid crystal (LC) molecules within these random wrinkles can be analyzed using a wave plate inserted at 45° on the bottom polarizer of the cross-polarizer at 530 nm. The unique molecular alignment of the random wrinkles produces red and blue interference colors that appear at random positions, as shown in Fig. 1b. Moreover, the random wrinkles PUF generates a specific output value due to the varying optical retardation dependent on the optic axis. These output values can be used to store information, and they can potentially be employed in various authentication methods, as illustrated in Fig. 1c.

### Fabrication and observation of the random wrinkles PUFs

**Fig. 2:**
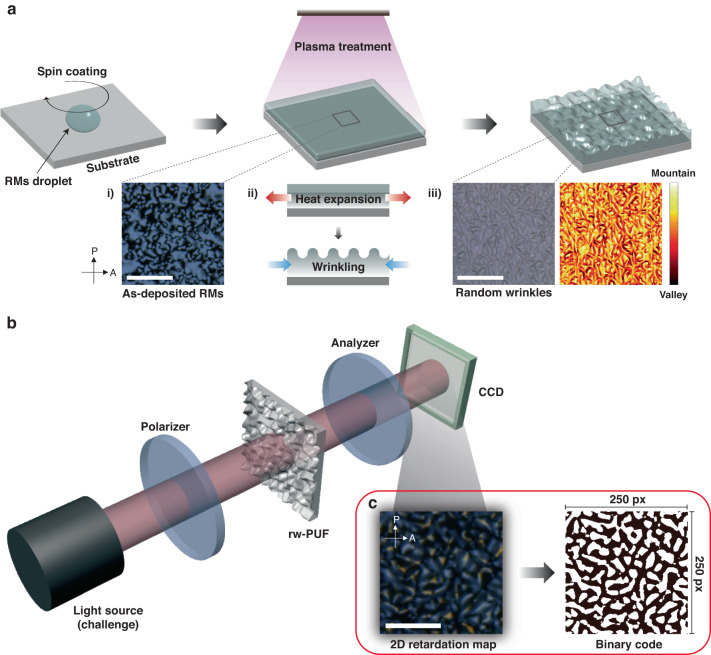
Fabrication procedure of rw-PUF. **a** The process of generating random wrinkles. When a liquid crystalline monomer of a reactive mesogen (RM) is spin-coated and cured using plasma, unique and non-replicable wrinkles are formed. (i) Polarized optical microscopy (POM) image showing the as-deposited RMs with random orientation. (ii) An illustration of the wrinkling process. (iii) Optical microscopy (OM) image showing the random wrinkles. **b** Schematic of the optical setup used for measuring pixelated birefringence of rw-PUF. **c** Schematic illustrating the conversion of a 2D retardation map to a binary code. All scale bar represents 50 μm

The fabrication process for random wrinkles shown in Fig. 2a involves several steps. First, a substrate with a pristine surface is spin-coated with a layer of reactive mesogen. Next, an O_2_ plasma is applied to induce photopolymerization, which primarily occurs on the surface of the reactive mesogen due to the low energy of the plasma. As a result, two distinct layers with different thermal expansion coefficients are formed, which can lead to the instability of the bi-layer structure under the heat of the plasma environment. The wrinkle direction aligns with the extraordinary-axis direction of the rod-shaped reactive mesogen, resulting in the emergence of random wrinkles^41,44–46^. Polarized optical microscopy (POM) images of the as-deposited reactive mesogens show the random orientation of RM molecules (Fig. 2a; (i)). The conversion of these molecules into random wrinkles due to plasma treatment was confirmed by optical microscopy (Fig. 2a; (ii) and (iii)). The optical setup used in the experiment (Fig. 2b) required two polarizers to measure the optical properties of the birefringent wrinkles. The arbitrary orientation and diverse amplitude of wrinkles produced a 2D retardation map that was detected by a CCD camera (1024 × 768) placed behind the analyzer. The detected 2D retardation map was finally converted into a binary code using a computer algorithm such as the Sauvola and Niblack method^47^ (Fig. 2c).

### Numerical analysis of the essential performance of random wrinkles PUF

**Fig. 3:**
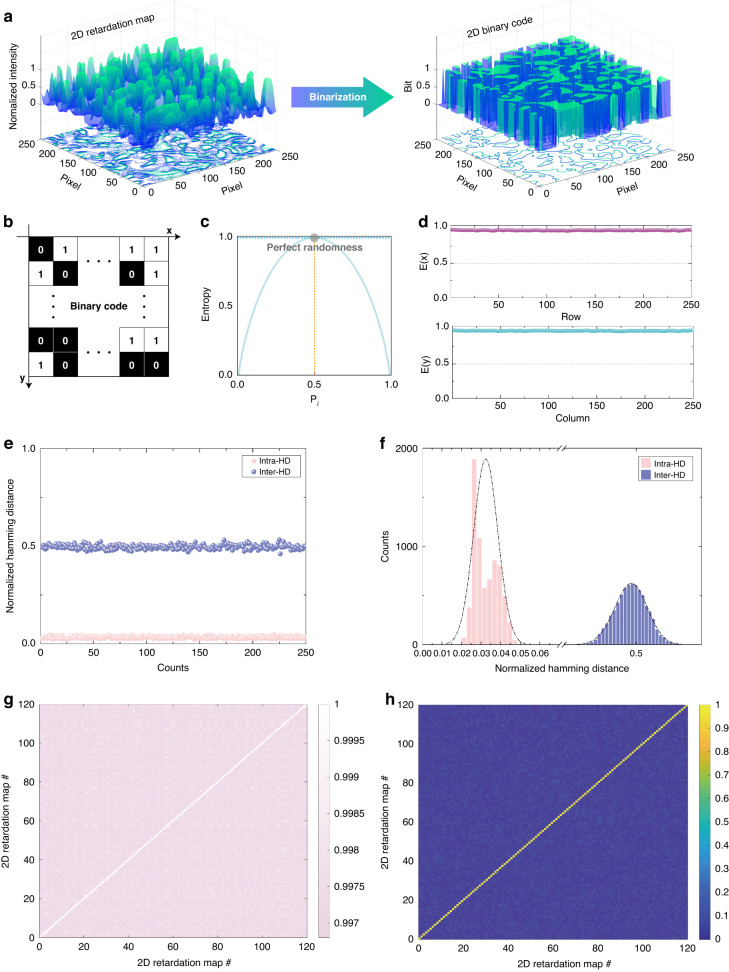
Unclonable characteristic of rw-PUF. **a** The Binarization process to convert from a grayscale 2D retardation map to a binary code. The 2D binary code is 250 × 250 pixels converted by computer algorithms. **b** Schematic of 2D binary code from the 2D retardation map of rw-PUF. **c** Measured entropy for an event (${p}_{i}$) and a perfect random property could be observed. **d** Entropy values ($E(x)$ and $E(y)$) along the row and column axes, respectively. **e** Intra-Hamming Distances (HDs) were obtained by repeatedly measuring a 2D binary code 250 times for a single rw-PUF, while Inter-HDs were computed between 2D binary codes generated by 250 distinct rw-PUFs. **f** Probability density functions of intra-HDs and inter-HDs. **g** Correlation coefficients between 120 re-measured 2D retardation maps for a single rw-PUF. **h** Correlation coefficients of 2D retardation maps generated by 50 distinct rw-PUFs

The opto-physical properties of PUF are defined in ISO/IEC 20897-1 as randomness, robustness, and uniqueness^48^. Here, we analyze the performance of a 2D binary code based on rw-PUF (Fig. 3a). The retardation map, acquired using an optical setup, is converted into a 2D binary code by applying an adaptive threshold algorithm (Fig. 3b). To quantify the randomness of the random wrinkles PUF, the entropy (E) values along each row and column axis of the 2D binary code using the standard equation provided by

1 $${E}_{i}={-[p}_{i}{\log }_{2}{p}_{i}+\left(1-{p}_{i}\right){\log }_{2}(1-{p}_{i})]$$

where $i$ represents the row and column axis, and ${p}_{i}$ is the probability that the pixel is set to ‘1’ along each axis^49^. Deterministic event distributions result in lower entropy values, indicating a reduced degree of uncertainty associated with the corresponding probability distribution. Conversely, a uniform distribution leads to higher entropy. As demonstrated in Fig. 3c, the relationship between the probability of an event and its entropy is shown. For instance, in coin flipping, if a coin with only one side (front or back) is flipped, the probability of the front or back side is either ‘0’ or ‘1’, and hence the entropy is ‘0’. In contrast, in a standard coin-flipping scenario, the probability of obtaining either the front or back side is ‘0.5’, resulting in an entropy value of ‘1’, which denotes a truly random outcome. The measured entropy values for the row and column axes of the rw-PUF were found to be 0.9793 ± 0.03 and 0.9806 ± 0.02, respectively, as shown in Fig. 3d. These values are close to the ideal entropy value of ‘1’, signifying that the rw-PUF exhibits high levels of unpredictability and randomness. In order to differentiate rw-PUFs generated through the same fabrication process, evaluating their uniqueness and distinctness is essential. The Hamming distance (HD) was employed to quantify the level of variation among binary strings associated with distinct PUFs. For example, the hamming distance between ‘110010’ and ‘101011’ is 3^50^. The intra-HD values, which represent the differences between identical PUFs, should ideally be zero, but they may exceed zero due to external factors such as temperature and ambient light.

In contrast, the inter-HD values, which correspond to variations between different PUFs, should ideally be 0.5 to ensure unique identification. To evaluate the distinctness of the rw-PUF, we measured HD values using a binary code obtained through an adaptive threshold algorithm applied to a 2D retardation map. The results showed that when the same rw-PUF was measured 250 times, the average and standard deviation of intra-HD values were 0.022 and 0.006, respectively. This proximity to the ideal value of zero indicates that the rw-PUF is reliable. However, a non-zero value due to CCD noise, fluctuations in light source, and optical setup vibrations during measurement may exist, causing slight variations. Upon measuring 250 different rw-PUFs, the results demonstrated uniqueness with an average inter-HD of 0.497 and standard deviation of 0.012, indicating that each PUF has a value that can be distinguished from others. This result is close to the ideal value of 0.5 for inter-HD, signifying the high uniqueness of the rw-PUF. Moreover, the inter-HD and intra-HD exhibit a narrow Gaussian distribution region ranging from 0.485 to 0.51 and 0 to 0.03, respectively, as shown in Fig. 3f. Additionally, we employed Pearson’s correlation formula to determine the correlation coefficients (CC) between the 2D retardation maps, which verified the uniqueness and robustness of the rw-PUF^51^. The CC value is calculated as follows:

2 $${{CC}}_{{XY}}=\frac{{\sum }_{i}^{n}(X-{X}^{{\prime} })(Y-{Y}^{{\prime} })}{\sqrt{{\sum }_{i}^{n}{\left(X-{X}^{{\prime} }\right)}^{2}}\sqrt{{\sum }_{i}^{n}{\left(Y-{Y}^{{\prime} }\right)}^{2}}}$$

Here, *X* and *Y* represent the intensities of the n_th_ pixel in the 2D retardation map, where n depends on each axis’s pixel quantity. *X*′ and *Y*′ represent the corresponding mean intensities. The intra-CC value was measured to be approximately 99% (~98.7%) on average, which is close to the ideal value of 1, indicating a strong correlation between the measured values of identical PUFs. The inter-CC value should ideally be zero to demonstrate dissimilarity among rw-PUFs. The inter-CC values of the 120 different rw-PUFs showed a <4% similarity at 3.41, confirming the uniqueness of the random wrinkles PUF.

### Adaptive threshold binarization

Image processing is an essential component of computer vision, where images are transformed to extract helpful information for interpretation and analysis. Binarization is necessary because it simplifies image data and reduces complexity, making it easier to process and analyze. By converting the image into a binary format, it is possible to enhance the desired features, making it easier to analyze the image. Binarization converts a grayscale or color image into a binary image by assigning a fixed threshold value to each pixel. A pixel value below the threshold is assigned a value of 0 (black), while a value above the threshold is assigned a value of 1 (white). Several binarization techniques include global thresholding, local thresholding, and adaptive thresholding. Global thresholding applies a fixed threshold to the entire image, while local thresholding applies different thresholds to different parts of the image^52^. Adaptive thresholding is an advanced technique that extends the concept of local thresholding. In this method, the threshold value for each pixel is dynamically determined based on the specific characteristics of its local neighborhood^53,54^. The adaptive thresholding technique calculates a specific threshold value for each pixel by considering the local intensity values. It offers increased resilience to changes in lighting and contrast compared to global and local thresholding techniques, where such variations can influence the threshold value.

**Fig. 4:**
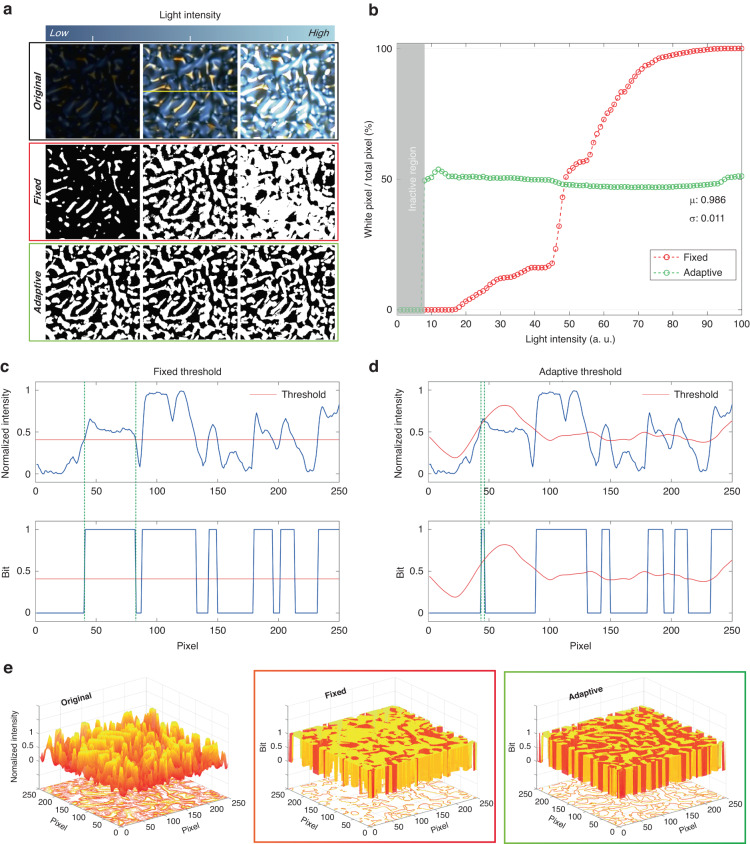
Adaptive binarization for PUF. **a** Comparison of PUF codes, including the original image, binary code comparison using fixed threshold, and adaptive threshold based on ambient light. **b** Variation in the ratio of white pixels with respect to ambient light intensity using different thresholding methods. When using the adaptive threshold method, the signals of 0 and 1 are adjusted to ~50%. **c**, **d** Comparison of binary codes using different thresholding methods. The red line represents the threshold that distinguishes between 0 and 1. **e** Visualization of a 2D intensity map showing the binary code obtained through the fixed threshold (red box) and the adaptive threshold (green box)

Additionally, it can retain intricate details and edges in the image, which may be lost during global thresholding. The threshold value for each pixel is determined by calculating the average intensity of the surrounding region on a pixel-by-pixel basis. If the intensity value of the center pixel is less than *T*% of the average, it is assigned a value of 0 (black); otherwise, it is assigned a value of 1 (white). *T* is a constant and is utilized to adjust the threshold value^54^. Optical PUFs, such as rw-PUF, are susceptible to variations in light intensity, making it essential to establish suitable threshold values during the binarization process. The resulting outcomes can differ significantly based on the chosen threshold. Figure 4a shows the binarization results of the rw-PUF under different light intensities. When applying a fixed thresholding method, the brightness of the resulting binary image varies depending on the original image and the intensity of the light. On the other hand, adaptive thresholding yields consistent outcomes, irrespective of the light brightness. As the brightness of the image increases, the fixed thresholding method produces an increasing number of white pixels in the 2D binary code.

In contrast, with adaptive thresholding, the number of white pixels remains almost constant, regardless of the brightness level. However, when the brightness is exceedingly low (~7%, or 1.225 mW cm^−2^), the binary process may not work, as it cannot be recognized. Additionally, although the threshold value may slightly fluctuate with changes in light intensity, the proportion of white pixels in the binary code remains relatively stable. The average and standard deviation of the results obtained through adaptive thresholding remains at 0.986 and 0.011, respectively, as shown in Fig. 4b. The binarization result of the pixel corresponding to the yellow line in Fig. 4a was measured using the threshold value. The green lines in both graphs were compared, and it was found that fixed thresholding generates binary images differently for each image intensity distribution since it identifies unnecessary pixels as either 0 or 1 (Fig. 4c). On the other hand, adaptive thresholding establishes an appropriate threshold value based on the image brightness, resulting in the extraction of the same results even with alterations in image brightness (Fig. 4d). Figure 4e illustrates a 3D graph to compare easily the binary code obtained when using the original image with high light intensity with fixed and adaptive threshold values. If a fixed threshold is employed (red box), most of the pixels may be assigned a value of 1 due to the high brightness of the original image. However, an adaptive threshold can yield more precise results (green box), as it can dynamically adjust the threshold value throughout the image. Note that, after converting wrinkles formed by RMs coated at speeds of 1000 and 7000 rpm into 2D binary codes, we observed a distinctive difference in the distribution of the area where light is transmitted (Fig. S3). Concerns were raised about potential variations in uniqueness and randomness based on the density of the white distribution area. However, it is noteworthy that the entropy value within the same region was measured to be close to 1, consistent with the existing rw-PUF. Therefore, this problem appears negligible since applying an adaptive threshold scales white pixels to 50% of the total pixels.

### Voxelization of 2D retardation maps

**Fig. 5:**
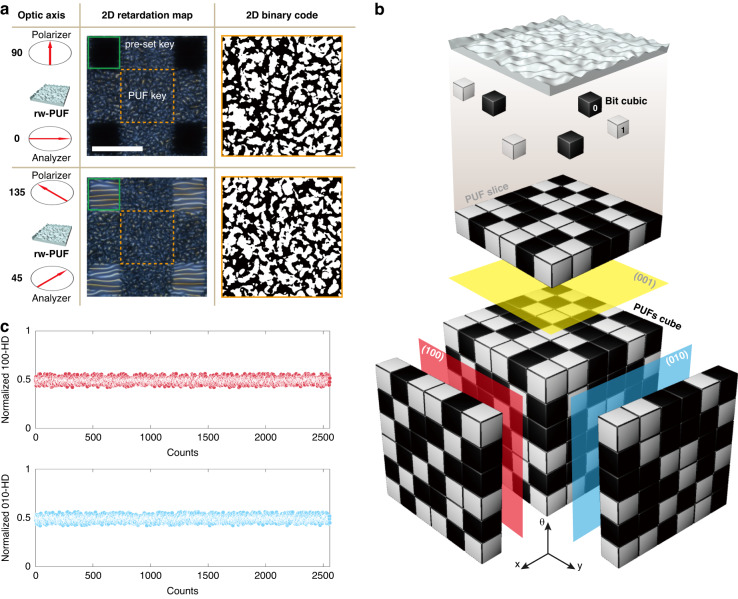
PUFs cube. **a** Observation of single rw-PUF depends on the optic axis of the crossed polarizer. The pre-set key is a wrinkle with a constant period to confirm crossed polarizer angle to sample. The scale bar is 50 μm. Binary code of the PUF key when the crossed polarizer angle to the sample is 0° (pre-set key = black) and 45° (pre-set key = white). **b** Schematic of PUFs cube reconstructed from the 2D rw-PUF slices. Various parameters, including the observation angle of the 2D PUF, can restructure the PUFs cube. **c** HDs between PUF slice generated by PUFs cube (100) direction and (010) direction with one rw-PUF

By utilizing 2D retardation mapping in rw-PUF, we introduce a novel technique for encoding 2D binary code data in a 3D PUF through simple polarization manipulation. Birefringence, the critical optical property of LCs, plays a significant role in this process. Birefringence denotes the variation in refractive index between directions perpendicular and parallel to the director axis of LCs. The director axis refers to the alignment direction of LC molecules, while the formation of wrinkles occurs parallel to the long-axis direction of these molecules^40^. When an LC layer is placed between crossed polarizers, the polarization of the incident light is rotated by an angle determined by the birefringence of the LC and the thickness of the layer. However, when the orientation of the LC molecules within the wrinkles is random, the resulting birefringence becomes random as well, causing the light to either not transmit or transmit only locally. Consequently, the transmitted light becomes partially polarized, and the polarization state depends on the orientation of the LC layer in relation to the polarizer. As the cross-polarizers rotate in relation to the wrinkles, the polarization of the transmitted light undergoes changes, leading to corresponding variations in the observed image. In other words, the orientation of the wrinkles in relation to the crossed polarizers generates a new 2D retardation map. Figure 5a shows the retardation maps and binary codes of different optic axes in one rw-PUF. This rw-PUF consists of wrinkles with a uniform period that act as a pre-set key to determine the optic axis and a reading area that captures a 2D retardation map. The retardation maps of the reading area were compared by setting the alignment key direction of the wrinkles at 0° (black) and 45° (white) relative to the polarizer. As a result of analyzing the entropy and HD of the two binary codes, it was confirmed that the two codes are random, and each has uniqueness (Figs. S2 and S4). This was not merely the result of physically rotating an rw-PUF sample, but rather a new 2D binary code was generated by birefringence due to the random orientation of LC molecules. The 2D retardation map data of rw-PUF can be voxelized in three dimensions (3D) along the optic axis (Fig. 5b). Each binary data point in the 2D binary code is converted into a “bit cubic,” which is then combined to form a “slice” of a 3D data structure. A 3D cube, representing voxel-based data, is partitioned into regular grids of equally sized cubes. Each voxel contains a value or a set of values representing a specific physical or mathematical property of an object within a specific region of space. In the case of rw-PUF, these values can represent the retardation map data based on the optic axis.

Additionally, an interesting observation was made; the slices in the *x*- and *y*-axis directions of the PUFs cube stacked according to the change in the optic axis, each slice becomes a unique two-dimensional data, which can be expressed as a Miller index. The Miller index is a notation system used in crystallography to describe the orientation of crystal faces and planes within a crystal lattice. It is a three-digit numerical representation of the orientation of a crystal face, determined by the intercepts of the face with the crystallographic axes. In the PUFs cube, the Miller indices are denoted as (*x*, *y*, *θ*), where x and y are the row and column in the 2D binary code, respectively, and θ designates the optical axis. We analyzed PUFs cube faces in the directions of the x and y corresponding to the Miller indices (100) and (010), respectively. Here, knowing the data of one plane has the potential to predict the other two planes easily. However, depending on the orientation of the cube data axis, the data on all planes can be completely different. If unauthorized access is attempted to PUF cube data, it is impossible to hack because the orientation of the initially set axis is unknown. Figure 5c illustrates the obtained HD values of the (100) and (001) slices of the PUFs cube. The PUFs cube used in the experiment comprised a 2D retardation map, which was measured with an increasing value of *θ* by 5. The 2D binary code was then converted to a resolution of 72×72 pixels to ensure the equivalence of the three axes of the PUFs cube. The HD of 72 PUF slices in each direction was measured, and the obtained values were found to be close to the ideal value of 0.5. This confirmed that each PUF slice possessed a unique value. The information storage capacity of the PUFs cube can be calculated using the formula N = 2*λ*/*θ*, where N represents the storage capacity, *λ* represents the number of PUF slices in each direction, and θ represents the increment of the optic axis rotation. To determine the actual storage capacity of the PUFs cube, we conducted experiments where we varied the optic axis rotation from 0 degrees to 360 degrees and analyzed the results (Movie S2). Our analysis revealed that the PUFs cube in the current system could store up to 2*λ*/5 (N = 72) data (Fig. S5).

### Polyhedron authentication method

**Fig. 6:**
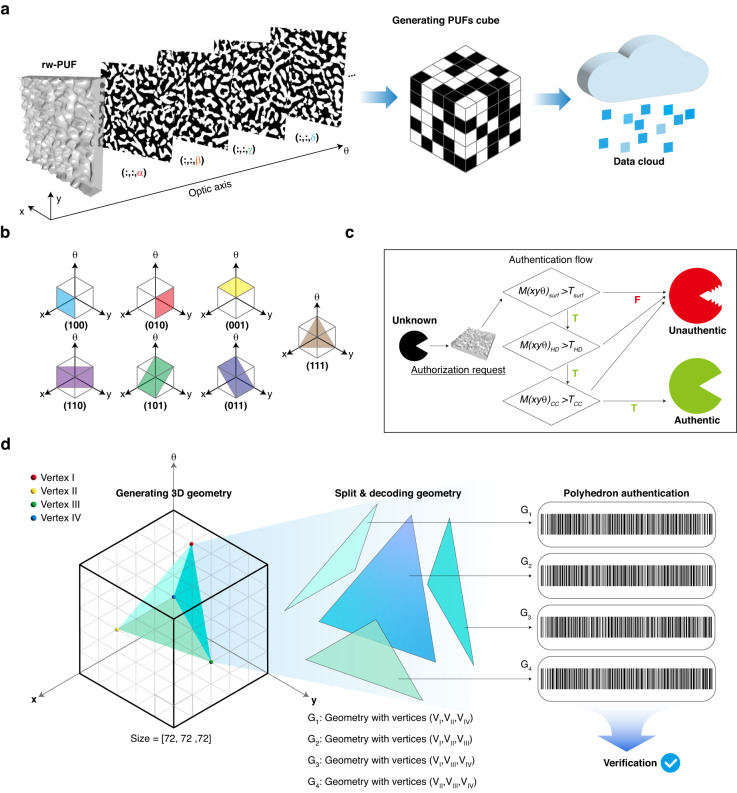
Polyhedron authentication system through the PUFs cube. **a** Illustration of generating a PUFs cube obtained from rw-PUF depending on the optic axis (α, β, γ, δ) and storing in a data cloud. **b** An example of a Miller index capable of extracting from PUFs cube. **c** The authentication flow of rw-PUF in the authorization request. Authorization can only be granted if multiple authentication codes are successfully verified. **d** A multi-dimensional authentication application using PUFs cube, and verification is performed through geometric vertices extracted through different Miller indexes

The proposed PUFs cube is stored in the cloud, enabling convenient authentication through polarization manipulation (Fig. 6a). The PUF data is also available in various Miller index forms, stored as three-dimensional cubes (Fig. 6b). This enhances the unpredictability of the PUF and strengthens its protection against unauthorized access. Ensuring system availability, integrity, and the protection of sensitive information are critical to authentication. Figure 6c shows a straightforward process for the physically random wrinkle-based PUFs step-by-step verification method. Upon receipt of an authentication request from an unknown PUF, a meticulous verification protocol is initiated to assess the randomness, uniqueness, and robustness of the PUF. This protocol begins with a physical surface scan and compares the obtained data with the stored data cloud, enabling discrimination between genuine and counterfeit PUFs. We introduce a novel authentication technique, the ‘3D polyhedron authentication method’, which utilizes a PUFs cube created from utterly random and distinct rw-PUFs, as shown in Fig. 6d. For example, specify four vertices in a 3D PUFs cube represented by a coordinate system (i.e., V_I_, V_II_, V_III_, V_IV_). The four specified vertices give rise to four faces, creating a triangular pyramid shape (i.e., G_1_, G_2_, G_3_, G_4_) in accordance with the *Euler* characteristic. Also, the number of 3D shapes that can be formed using four vertices can be defined as follows: ${pCr}=p!/\left[r!\left(p-r\right)\right]!$ Here, *p* represents the maximum number of vertices that can be selected, and *r* represents the number of vertices to be selected. For example, if a PUF cube of size 72 × 72 × 72 is formed using any 4 vertices to form the shape, the p is 73^3^ = 389,017 ranging from (0,0,0) to (72,72,72). Therefore, the number of possible combinations to form a shape using four random vertices is approximately 9.54236×10^15^. Also, the number of possible combinations increases when we consider other polyhedrons, as seen in Euler’s polyhedron formula, which includes cubes, octahedrons, and dodecahedrons, among others. Each polyhedron’s unique configuration and varying number of vertices contribute to a larger diversity of possible combinations, expanding the scope and complexity of the shapes that can be formed using different sets of vertices. This further underscores the richness and versatility of the system’s degree of freedom in generating diverse 3D shapes. In our approach, the binary code of each face is reconstructed into a one-dimensional code using our proprietary algorithm. The identification process is deemed successful if each code passes validation against the data cloud. This methodology aligns with the fundamental principles of security found in locks and keys. Just as inserting the correct key aligns the notch with the pin and unlocks the mechanism, inserting an incorrect key that does not match the pin will not unlock it.

Likewise, each G (face) functions as a notch in the key, while the data cloud serves as the pin in our approach. A binning process was performed to convert 2D binary code into 1D code (Fig. S6). The 1D code formed by the binary value of the polyhedral face, with an entropy close to 1, demonstrates an unbiased distribution towards ‘0’ or ‘1’, rendering it highly secure. The proposed authentication method exhibits versatile applicability, encompassing secure communication, anti-counterfeiting measures, and access control systems, among others.

## Discussion

This study presents a ground-breaking approach to information encoding using a two-dimensional random wrinkles PUF within a three-dimensional directional framework. By leveraging the complex surface structure formed between crossed-polarizers, effective light scattering and diffraction are induced, resulting in the generation of a two-dimensional retardation map. Thorough theoretical analysis has confirmed the inherent properties of the binary codes derived from random wrinkles PUFs, demonstrating their robustness, randomness, and uniqueness, thereby establishing their suitability for optical PUF applications. Moreover, the system enables the extraction of multiple unclonable functions from a single PUF through optic axis manipulation, which is then integrated into a cohesive three-dimensional code representation. Using a PUFs cube in this manner shows promising potential for enhancing security in continuous authentication systems, thereby elevating the overall level of protection and reliability.

## Materials and methods

### Fabrication of random wrinkles

Soda-lime slide glass (HSU-1000412, Marienfeld) was sonicated in acetone (400Series, Hwashin Tech Co., Ltd.), rinsed with distilled water, and dried using a nitrogen flow (N_2_, 99.95%) to prepare the substrate. A commercial solution of reactive mesogens (RMS03-001C, Merck KGaA, Darmstadt, Germany) was used as the active material for wrinkles fabrication. The reactive mesogens consist of 4-(6-acryloyloxyhexyloxy)-benzoic acid (4-cyanophenyl ester), 4-(3-acryloyloxypropyloxy)-benzoic acid 2-methyl-1,4-phenylene ester, 4-(6-acryloyloxyhexyloxy)-benzoic acid-(4-methoxyphenyl ester), and 2-methyl-1,4-phenylene-bis[4-(6-acyloyloxyhexyloxy)benzoate]. This solution was spin-coated (SF-100ND, Rhabdos) onto the glass substrate at a speed of 3000 rpm for 30 seconds. The RM-coated glass was exposed to oxygen plasma (CUTE, Femto Science Co.) for 1 minute at an RF power of 50W, using 20 sccm oxygen as the reactant gas and 7 sccm argon as the carrier gas. (Fig. S1)

### Optical measurement setup (characterization)

2D retardation maps were obtained using a polarized optical microscope (DM750P, Leica) with a multi-color CCD camera (MC170, Leica), which has a 1024×768 pixels resolution. An automatic precision stage (TI2-S-HIU, Nikon) was used to align each measurement to the correct position. The transmittance intensity was measured using a power meter (S120VC, Thorlabs). The maskless lithography setup used in our previous work was used to fabricate pixelated wrinkles corresponding to the pre-set keys in Fig. 5a.

### Image binarization and analysis process

The 2D binary codes were converted with an in-house-developed MATLAB (MathWorks) algorithm. The 2D retardation maps obtained random wrinkles were filtered and converted according to the light intensity of original images using “rgb2gray”, “otsuthresh”, and “adaptthresh” functions. During the binarization process, the T value was customized. To measure the entropy value of the converted 2D binary code, we analyzed using 250 samples (Fig. 3d and Movie S1). To measure the Inter Hamming distance, 250 different samples were compared and analyzed, and one sample was re-measured 250 times to measure the Intra Hamming distance (Fig. 3e, f) using “xor” and “nnz” functions. To measure the coherence efficiency, 120 different samples were analyzed using “corr2” functions.

### Supplementary information

Supplementary Material

Movie S1. Comparison of inter-PUF pattern

Movie S2. PUF pattern in the optical axis
