# Supplementary material for: Voxelated opto-physically unclonable functions via irreplicable wrinkles
Source: Light Sci Appl. 2023 Oct 3;12:245. doi: 10.1038/s41377-023-01285-1 (PMC10547705; doi:10.1038/s41377-023-01285-1)
Supplement: Supplementary file 1 — Supplementary Material [file 41377_2023_1285_MOESM1_ESM.docx]

Supplementary Information for

Voxelated Opto-Physically Unclonable Functions via Irreplicable Wrinkles

Kitae Kim^1^, Se-Um Kim^2^, Moon-Young Choi^1^, Mohsin Hassan Saeed^3^, Youngmin Kim^4^, and Jun-Hee Na^1,3^*

^1^Department of Convergence System Engineering, Chungnam National University, 99 Daehak-ro, Yuseong-gu, Daejeon 34134, Republic of Korea.

^2^Department of Electrical and Information Engineering, Seoul National University of Science & Technology, 232 Gongneung-ro, Nowon-gu, Seoul 01811, Republic of Korea.

^3^Department of Electrical, Electronics, and Communication Engineering Education, Chungnam National University, 99 Daehak-ro, Yuseong-gu, Daejeon 34134, Republic of Korea.

^4^Hologram Research Center, Korea Electronics Technology Institute, World Cup buk-ro 54-gil, Mapo-gu, Seoul 03924, Republic of Korea.

^*^Corresponding author: Prof. Jun-Hee Na (email: [junhee.na@cnu.ac.kr](mailto:junhee.na@cnu.ac.kr))

**Supplementary Figures**


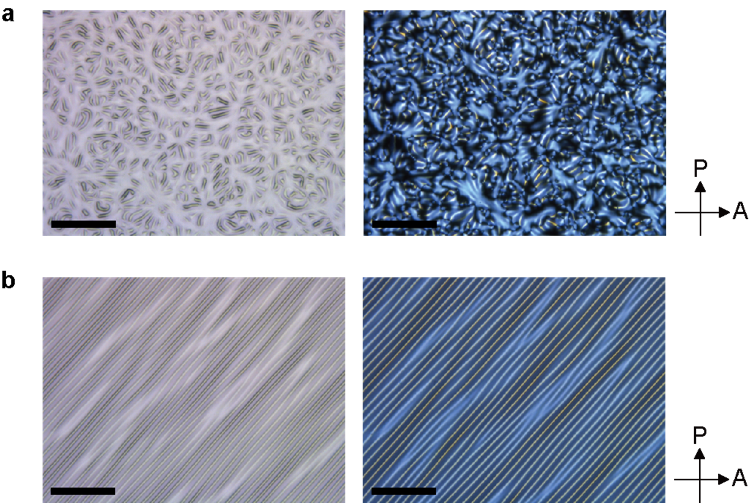


Fig. S1. Observation of (a) random and (b) periodic wrinkles using an optical microscope and polarizing optical microscope.


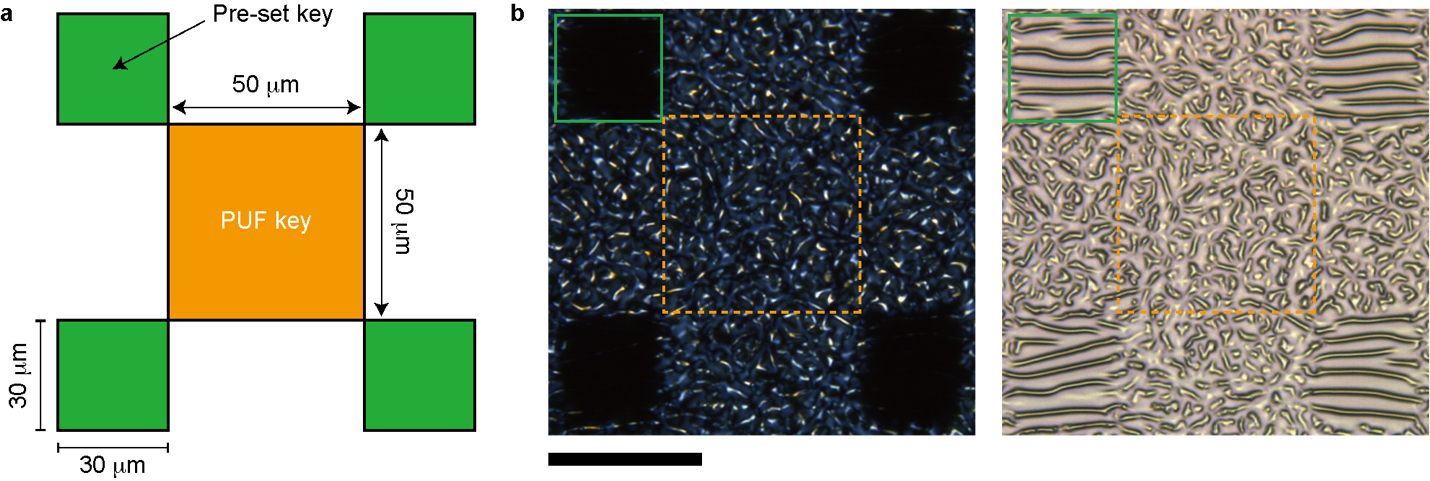


**Fig. S2. Schematic of the rw-PUF.** Schematic and observation of the rw-PUF. (a) Schematic representation of the rw-PUF showing the PUF key area (orange box) and the pre-set key area for optical axis verification (green box). The PUF key has a size of 50 μm by 50 μm, while the pre-set key has a size of 30 μm by 30 μm. The rw-PUF incorporates an uneven waveform surface placed between cross-polarizers, which induces light scattering and diffraction, resulting in the generation of a two-dimensional retardation map. (b) Visual observation of the rw-PUF using a polarizer (left) and without a polarizer (right). The scale bar provided indicates a length of 50 μm.


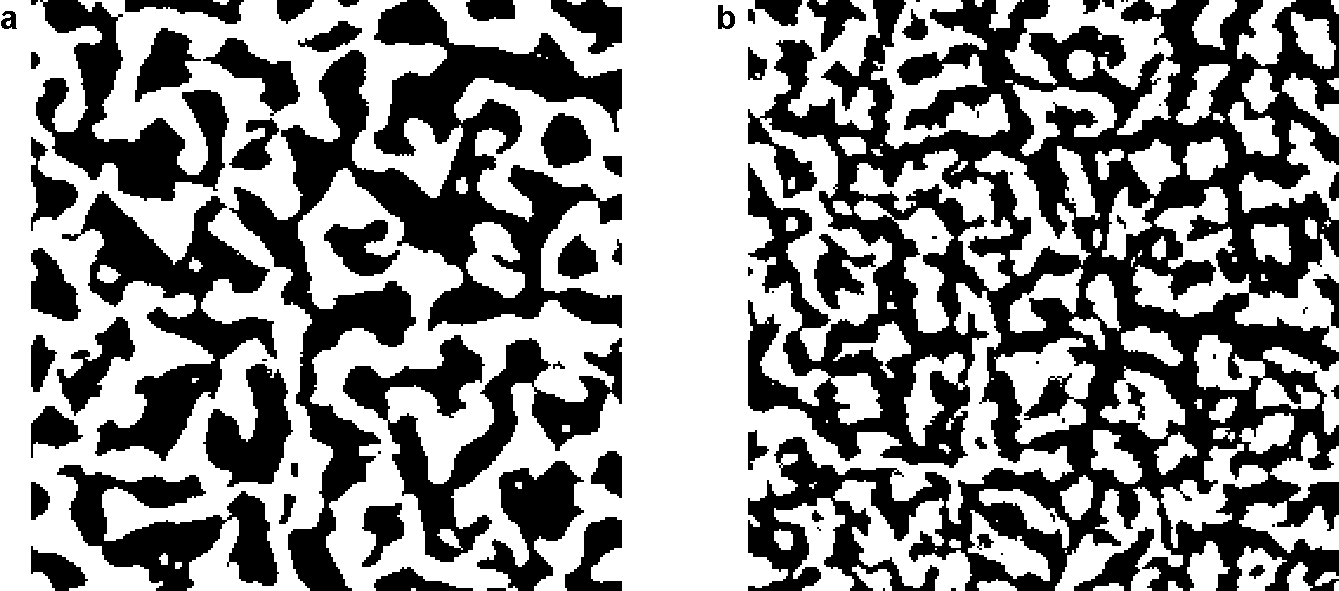


**Fig. S3. 2D binary code according to thickness.** Conversion of wrinkles formed by coated RMs at speeds of (a) 1000 rpm and (b) 7000 rpm into 2D binary codes.


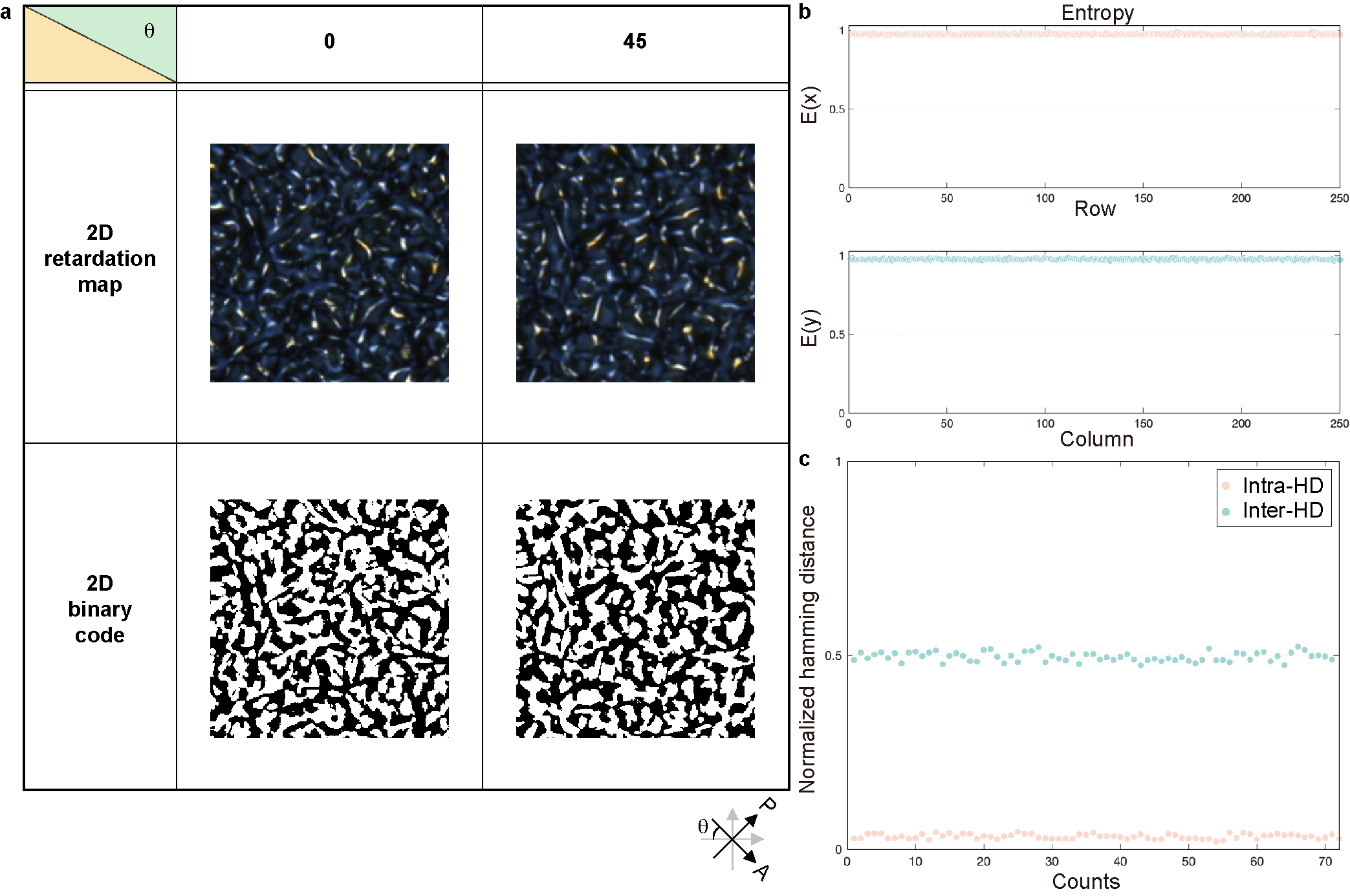


Fig. S4. 2D retardation map and binary code of rw-PUF. (a) The 2D retardation map and binary code variations of the rw-PUF are shown for two optical axis positions: 0 and 45 degrees. The retardation map visualizes the spatial distribution of retardation values, while the binary code represents the digital representation of the retardation map using binary values (0 or 1). (b) The entropy of the 2D binary code obtained from the rw-PUF is evaluated in the case of an optical axis position of 45 degrees. Entropy measures the randomness or uncertainty in the binary code, with higher entropy indicating a more unpredictable pattern. (c) The Hamming distance of the 2D binary code obtained from the rw-PUF is analyzed with an optical axis position of 45 degrees. Hamming distance quantifies the dissimilarity between two binary sequences, measuring the number of positions at which they differ. A lower Hamming distance suggests a higher similarity between binary codes.

**
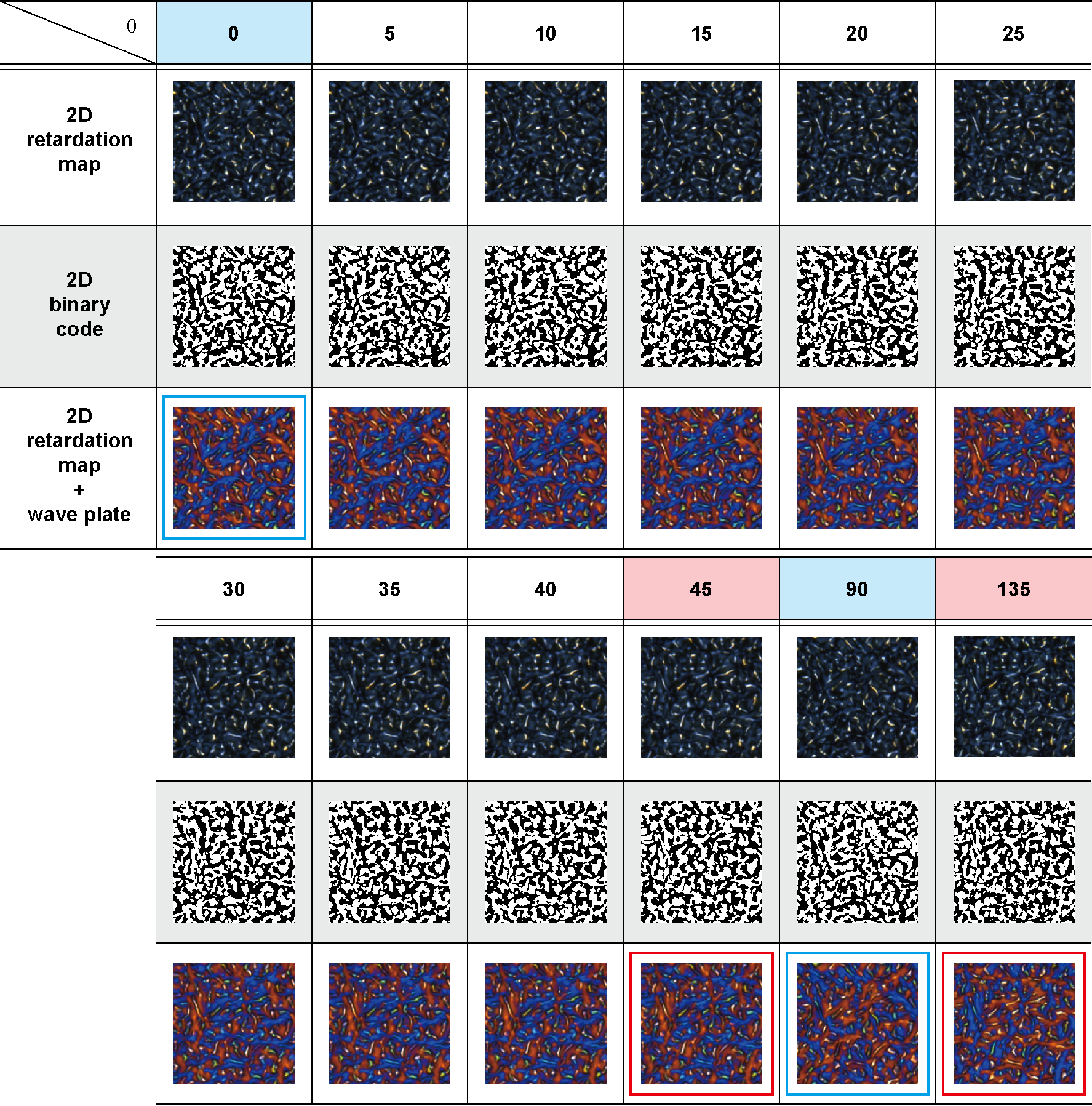
**

**Fig. S5. 2D retardation map and binary code for multiple optical axes. Comparison of the changes in binary code and 2D retardation map of the rw-PUF with varying optic axis and pre-set key values between cross-polarizers. The measured 2D retardation map may appear similar when the pre-set key is black (0, 90, 180, 270 degrees) or white (45, 135, 225, 315 degrees) but can be verified to be different using a wave plate. Observations reveal that the similarity between the 2D retardation map and binary code is evident when the optic axis is at 0 degrees and 90 degrees, but viewing through a wave plate shows an entirely different retardation map (blue box). The similarity is observed when the optic axis is at 45 degrees and 135 degrees, but a different retardation map is observed with a wave plate (red box).**

**
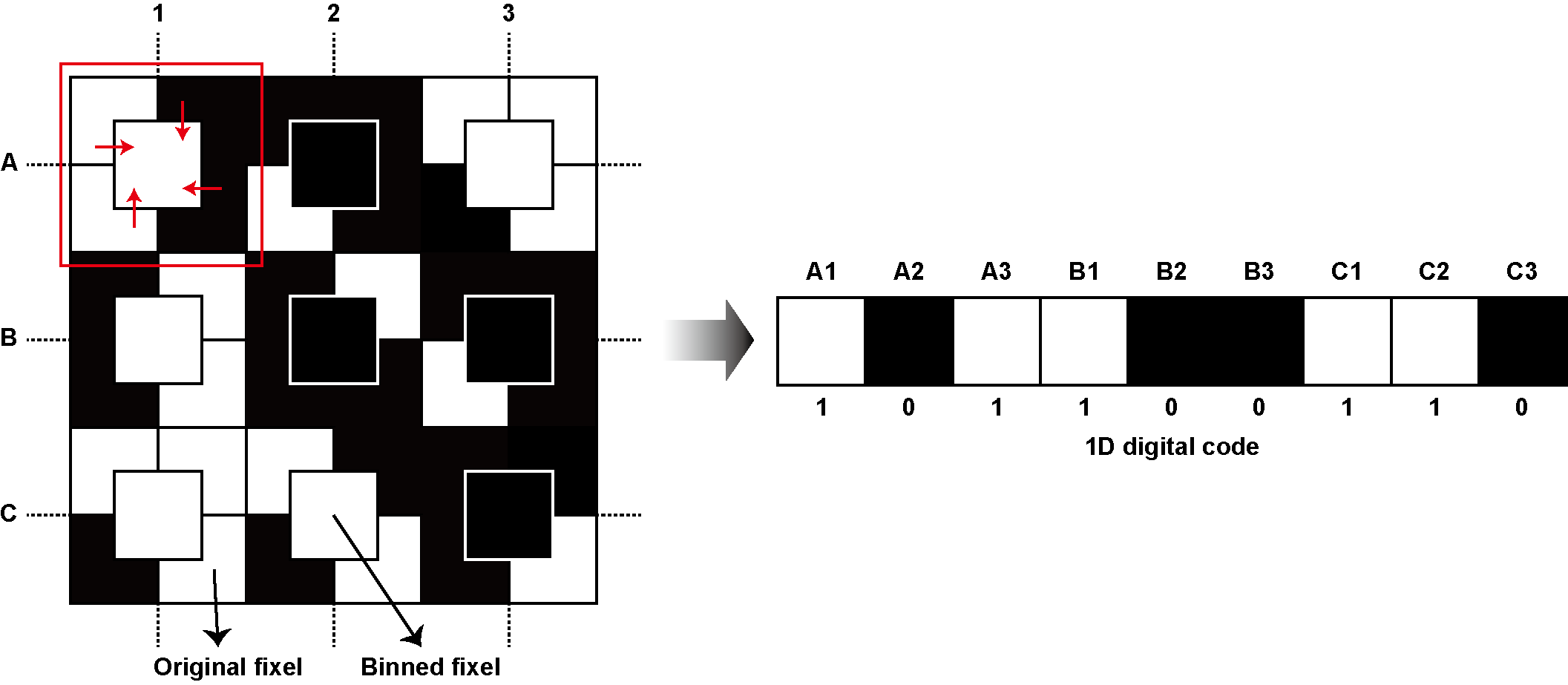
**

**Fig. S6. Binning process. An illustration of the binning process. The data of the 2D binary code is clustered in the same boundary unit. The binned data is reconstructed in a predetermined sequence and converted into a one-dimensional digital code.**

**Movies**

Movie S1. Comparison of the 2D retardation map and binary code for 100 random write physical unclonable functions (rw-PUFs)

Movie S2. 2D retardation map and binary code variations of a single rw-PUF corresponding to changes in the optical axis
